# Efficacy and adverse effects of transdermal fentanyl and sustained-release oral morphine in treating moderate-severe cancer pain in Chinese population: a systematic review and meta-analysis

**DOI:** 10.1186/1756-9966-29-67

**Published:** 2010-06-09

**Authors:** Qiong Yang, De-Rong Xie, Zhi-Min Jiang, Wen Ma, Yuan-Dong Zhang, Zhuo-Fei Bi, Deng-Lin Chen

**Affiliations:** 1 Department of Oncology, The Sun Yat-sen Memorial Hospital, Sun Yat-sen University, 107 west Yanjiang Road, Guangzhou, Guangdong, 510120, China

## Abstract

**Background:**

Previous meta-analysis suggested that transdermal fentanyl was not inferior to sustained-release oral morphine in treating moderate-severe cancer pain with less adverse effects. Now, we updated the data and performed a systematic review.

**Methods:**

Updated cohort studies on transdermal fentanyl and oral morphine in the treatment of cancer pain were searched in electronic databases including CBMdisc, CNKI, VIP, Medline, EMBASE and Cochrane Library. Primary end points assessed by meta-analysis were remission rate of pain and incidence of adverse effects. Quality of life was assessed by systematic review, which was the second end point.

**Results:**

32 cohort studies, which included 2651 patients, were included in present study. The remission rate in transdermal fentanyl group and sustained-release oral morphine group were 86.60% and 88.31% respectively, there was no significant difference [RR = 1.13, 95% CI (0.92, 1.38), P = 0.23]. Compared with oral morphine group, there were less adverse effects in terms of constipation [RR = 0.35, 95% CI (0.27, 0.45), P < 0.00001], nausea/vomiting [RR = 0.57, 95% CI (0.49, 0.67), P < 0.00001], and vertigo/somnolence [RR = 0.59, 95% CI (0.51, 0.68), P < 0.00001] in transdermal fentanyl group. Six of selected trials supported either transdermal fentanyl or sustained-release oral morphine improved QOL of cancer patients and one of them showed more patients got better QOL after sustained-release oral morphine transferred to transdermal fentanyl.

**Conclusions:**

Our study showed again that both transdermal fentanyl and oral morphine had the same efficacy in the treatment of moderate-severe cancer pain in Chinese population, but the former might have less adverse effects and better quality of life.

## Background

For patients with cancer, up to 70% suffered from pain caused by their disease or its treatment [1]. For patients with advanced cancer, pain was described as moderate-severe in approximately 40%-50% and as very severe in 25%-30% [2]. Because pain was an important symptom and occurred frequently in cancer patients, especially for moderate-severe cancer pain, relief of pain should therefore be seen as part of a comprehensive pattern of cancer care.

Since the 1980s, treatment of cancer pain was based on the WHO analgesic ladder. Strong opioids were classified at the highest step of the analgesic ladder. But studies of cancer pain control consistently revealed that up to half of patients received inadequate analgesia and 30% did not receive appropriate drugs for their pain [1]. In China, sustained-release oral morphine and transdermal fentanyl were strong opioids available for the treatment of moderate-severe cancer pain.

Fentanyl is a lipid soluble synthetic opioid, which can be delivered in a transdermal controlled systemic delivery formulation for up to 72 hours. Transdermal fentanyl was accepted to be an effective drug for treating moderate-severe cancer pain. Because it takes 12-24 hours for serum levels to stabilize after starting the patch or changing the dose, it was less flexible and suitable for patients with unstable pain. However, transdermal fentanyl may reduce the rates of some typical opioid-related adverse effects, particularly constipation [3]. In addition, transdermal fentanyl was conveniently administrated, which simplified the procedure of chronic pain treatment and improved the compliance for using the analgesic.

Three systematic reviews of European and American literatures suggested both transdermal fentanyl and sustained-release oral morphine could effectively control moderate-severe cancer pain, but some adverse effects (mainly constipation) seemed to favor transdermal opiates in the preference of patients with moderate-severe cancer pain [4-6]. Our previous meta-analysis of 12 Chinese literatures also found similar result [7]. Since then, many clinical trials were conducted again in China to compare the two drugs [8-39]. Therefore, we updated the data and re-performed a systematic review of all related literatures to evaluate efficacy and adverse effects of transdermal fentanyl and oral morphine treating moderate-severe cancer pain in Chinese population.

## Methods

### Search Strategy

Two authors independently performed a systematic review of electronic databases including Chinese Biomedical Literature Analysis and Retrieval System (CBMdisc), China National Knowledge Infrastructure (CNKI), Chongqi VIP Information (VIP), Medline, EMBASE and Cochrane library. The following keywords were used in the search: transdermal fentanyl, morphine, sustained-release morphine, Durogesic, MS Contin, Morphine Hydrochloride-Southwest Pharm. In addition to the online search, references from original articles also were scanned to capture missing clinical trial data that met our inclusion criteria. All papers comparing transdermal fentanyl with sustained-release oral morphine (MS Contin or Morphine Hydrochloride-Southwest Pharm) were examined. No language restrictions were applied. The deadline of last search was December 31, 2009.

### Inclusion Criteria

#### Study design

Trials should be prospective cohort study, which were matched for sex, age, performance status, and type of tumor.

#### Study population

Patients were Chinese and suffered from moderate-severe cancer pain. In addition, patients who were eligible for trials didn't receive radiotherapy, chemotherapy or immunotherapy in 30 days prior to analgesics administration, and patients had no history of hypersensitive to opioid or opioid abuse. Patients had adequate hematological, renal, cardiac and hepatic function.

#### Interventions

The treatment arm received transdermal fentanyl (Durogesic), the control arm received sustained-release oral morphine (MS Contin or Morphine Hydrochloride-Southwest Pharm). The treatment duration was 15 days at least.

#### End Points

The primary end points were remission rate of pain and incidence of opioids-related adverse effects. The second end point was quality of life (QOL).

### Data Extraction

Two primary reviewers (QY and DRX) assessed all abstracts that were identified from the above-mentioned sources. Both reviewers independently selected trials according to inclusion criteria. Disagreements were resolved by consensus or by the third reviewer (ZMJ). Following data were requested: number of patients recruited, number of patients had remission, number of patients had non-remission, number of patients experienced constipation, number of patients experienced nausea and vomiting (nausea/vomiting), number of patients experienced vertigo and somnolence (vertigo/somnolence) and QOL from each trial.

### Assessment of Study Quality

We assessed all manuscripts that met the selection criteria for quality. Quality assessment was based on published checklists. The checklists were adapted from MOOSE standard, which include six measures in total: prospective study design, groups comparable on all important confounding factors, outcome assessed blind to exposure status, follow-up long enough for outcomes to occur (defined as over 15 days), relation between outcome and exposure appropriately measured, and appropriate statistical analyses used. The maximum quality score was 6 point [40,41]. The quality scores were showed in additional file 1.

### Statistical Analysis

The primary end points variables were defined as dichotomous data (e.g., remission rate of pain used variables as follows: the effective or the ineffective after treatment). We standardized the therapeutic results by obtaining the relative risk (RR). RR is defined as a ratio of risk of uncontrolled pain or adverse effects occurring in transdermal fentanyl group versus sustained-release oral morphine group. To test for heterogeneity among the trials, Cochran's χ^2 ^test was used. P-value of more than 0.05 for the χ^2^-test indicated a lack of heterogeneity across the studies, so pooled estimation of the RRs of each study was calculated by the fixed effects model. Otherwise, the random effects model was used. An estimate of the potential publication bias was carried out by funnel plot, in which the standard error (SE) of log RR of each study was plotted against its log RR. An asymmetric plot suggested a possible publication bias. All analyses were performed strictly with RevMan software (version 4.2.8, Cochrane). *P *value less than 0.05 was considered as significant in difference.

## Results

### Characteristics of selected trials

578 trials were examined in the preliminary review; 32 of them were considered eligible and included in the analysis. The data extracted from 32 trials were shown in additional file 1[8-39]. A total of 2651 cancer pain patients were treated in all selected trials, 1296 with transdermal fentanyl, and 1355 with sustained-release oral morphine. 30 of selected trials were included in the analysis of clinical efficacy; and 31, 31 and 28 of selected trials were included in the analysis of constipation, nausea/vomiting and vertigo/somnolence. Only 6 trials supplied data about QOL evaluated in different criteria [9,14,17,32-34]. Sustained-release oral morphine was Morphine Hydrochloride-Southwest Pharm in 8 of selected trials [8,16,19,25,27,29,32,33]. Trials were excluded from the analysis for one or more of the following reasons: uncorrelated, review, case report, no valid data, no followed-up time, and non-cancer pain. Trials applied either numerical rating scale or visual analogue scales for assessing cancer pain. The criterion of remission of cancer pain was described as follow. Five categories of pain relief: category 0, no remission (pain didn't release); category 1, mild remission (pain released one quarter); category 2, moderate remission (pain released a half); category 3, obvious remission (pain released three quarters); category 4, complete remission (pain disappeared). Pain can be controlled denotes that patients gain category 2 or above of pain relief. Pain can't be controlled denotes patients gain category 1 or below of pain relief. Toxicity profiles were reported according to the WHO's criteria. QOL was reported in different criteria, which based on different QOL scale.

### Remission Rate of Pain

2491 patients from 30 cohort studies, 1216 in the transdermal fentanyl group and 1275 in the sustained-release oral morphine group were included in the meta-analysis of clinical efficacy. Overall effect of remission rate of pain was analyzed by a fixed-effect model (fixed), because test for heterogeneity among the trials was not significant (p = 1.00). The remission rate in transdermal fentanyl group and sustained-release oral morphine group were 86.60% and 88.31% respectively, there was no significant difference [RR = 1.13, 95% CI (0.92, 1.38), P = 0.23]. More details were shown in Table 1 and the forest plot was shown in additional file 2.

**Table 1:** Comparisons between Transdermal Fentanyl and Sustained-release Oral Morphine

Endpoints	No. of patients/studies	RR (95% CI)^a^	P^b^	P_h_^c^
Remission rate	2491/30	1.13 (0.92, 1.38)	0.23	1.00
Constipation	2593/31	0.35 (0.27, 0.45)	< 0.00001	< 0.00001
Nausea/vomiting	2593/31	0.57 (0.49, 0.67)	< 0.00001	0.009
Vertigo/somnolence	2300/28	0.59 (0.51, 0.68)	< 0.00001	0.08

^a ^RR, relative risk; 95% CI, 95% confidence interval

^b ^p value of significance tests of RR = 1

^c ^p value of heterogeneity tests

### Adverse Effects

Data on main adverse effects was summarized in the additional file 1. Overall effect of constipation and nausea/vomiting were analyzed by a random-effect model (random), because test for heterogeneity among the trials was significant (p < 0.05). Compared with sustained-release oral morphine, pooled RR of constipation was 0.35 [95%CI (0.27, 0.45), p < 0.00001]; pooled RR of nausea/vomiting was 0.57 [95%CI (0.49, 0.67), p < 0.00001]. Overall effect of vertigo/somnolence was analyzed by a fixed-effect model (fixed), because test for heterogeneity among the trials was not significant (p = 0.08). Pooled RR of vertigo/somnolence was 0.59 [95%CI (0.51, 0.68), p < 0.00001] in patients used transdermal fentanyl. In short, transdermal fentanyl caused less adverse effects in comparison of sustained-release oral morphine in patients with moderate-severe cancer pain. More details were showed in Table 1 and the forest plots were shown in additional file 2.

### Quality of Life

Six of selected trials were included to systematic review of QOL [9,14,17,32-34]. Primary endpoints of QOL were appetite, sleep, activity of daily living, mental states, emotion, communication and interest. QOL was not pooled for meta-analysis because different QOL evaluation criteria were used. After review of these six trials, all the data from each trial supported either transdermal fentanyl or sustained-release oral morphine improved QOL of cancer patients. In trial of Pang et al., more patients got better QOL after sustained-release oral morphine transferred to transdermal fentanyl [34].

### Publication Bias Assessment

**Figure 1:**
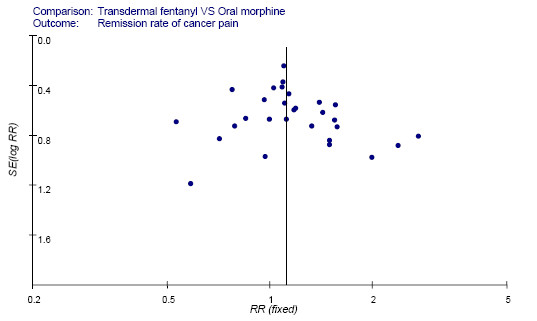
**Funnel plot of test for publication bias**. The vertical line represents the meta-analysis summary estimate, and the scatter represents single study. In the absence of publication bias, studies will be distributed symmetrically right and left the vertical line. logRR, natural logarithm of the RR; SE(logRR), standard error of the logRR.

In the funnel plot, the selected trials were plotted by the RR of the endpoints for meta-analysis and SE (logRR) as abscissa and ordinate. Small sample studies scattered widely at the bottom of the graph, while the spread narrowed for larger sample studies. Funnel plot was symmetrically distributed, and there was no influence of publication bias in our study (Figure 1).

### Sensitivity Analysis

Sensitivity analysis should be used to analyze stability of data when heterogeneity existed among selected trials. A single study involved in the present meta-analysis was deleted each time to reflect the influence of the individual data-set to the pooled RRs of constipation and nausea/vomiting, and the corresponding pooled RRs were not materially altered (data not shown).

## Discussion

Opioids were main drugs for managing pain according to WHO analgesic ladder. Oral morphine is generally accepted to be the drug of choice for maintenance therapy of moderate-severe cancer pain. But transdermal fentanyl is challenging the position because of its convenience, relative lower incidence of constipation and higher compliance of patients reported in clinical trials [42-44]. Clark et al and Tassinari et al in three meta-analyses reported two drugs were equally effective in improving the score of pain with less adverse effects for transdermal fentanyl [4-6].

In our meta-analysis, transdermal fentanyl and oral morphine were effective in controlling moderate-severe cancer pain. 86.60% patients with cancer pain would experience 50% or greater pain reduction by transdermal fentanyl, in contrast, 88.31% for oral morphine, but it didn't reach significant difference [RR = 1.13, 95% CI (0.92, 1.38), P = 0.23]. The result supported NCCN guideline (adult cancer pain-V.1.2009) that transdermal fentanyl and oral morphine were alterative drugs for maintenance therapy of stable moderate-severe cancer pain. In other words, both drugs were also effective in treating moderate-severe cancer pain in Chinese population, which might suggest both of opioids have no race choose.

Adverse effect and QOL might be more important indications for choosing drug when the therapeutic effect was similar between two drugs. In our meta-analysis, transdermal fentanyl caused less adverse effect compared with oral morphine, which the risk reduced 65% in constipation, 43% in nausea/vomiting and 41% in vertigo/somnolence. All reached significant difference (*P *< 0.05). Constipation caused by opioids was irreversible and even severely influenced QOL, but other adverse effects were reversible after 1-2 weeks use of opioids. Therefore, constipation is a common reason for patients refusing to continue to use opioid analgesics. Because of the less adverse effects, especially for constipation, transdermal fentanyl might be easier to improve QOL. In present study, 6 trials reported data on QOL and showed either transdermal fentanyl or sustained-release oral morphine improved QOL of cancer patients [9,14,17,32-34]. Especially, one of trials supported more patients got better QOL after sustained-release oral morphine transferred to transdermal fentanyl [34].

Cost effectiveness was not an endpoint in the present systematic review, but it was a valuable index to evaluate a drug for clinical use. 2 out of selected trials reported data about cost effectiveness that transdermal fentanyl had higher expenditure to control certain pain than oral morphine [35,36]. However, we should keep in mind that cost effectiveness was affected by many factors in fact and only 2 out of 32 trials reported data about cost effectiveness when we concluded cost effectiveness was higher in transdermal fentanyl.

Similar with European and American data [4-6], our data also showed that both transdermal fentanyl and sustained-release oral morphine were effective in treating stable moderate-severe cancer pain in Chinese population with less adverse effects for transdermal fentanyl. However, two differences should be pointed out. First, QOL was only analyzed in our study, and data suggested that transdermal fentanyl potentially improved QOL of cancer pain patients and resulted in better compliance compared with oral morphine. Second, more patients were included in the present systematic review and all patients were Chinese.

To explain the results reasonably, several issues should be considered as follow. First, the data source was extracted from abstracted data and not individual patient data (IPD). In general, an IPD-based meta-analysis would give a more robust estimation for the association; therefore, we should interpret the results with care, especially for a positive result. Clearly, further investigations using IPD should be conducted to examine the main end points. Second, all selected trials were cohort studies, which is not most suitable clinical trial to explore the difference of two drugs. Third, heterogeneity existed among the trials when pooled analysis of adverse effects (constipation and nausea/vomiting), fortunately, the data was not materially changed in sensitivity analysis. Fourth, side effects seemed to be lower in our selected trials compared with clinical practice. We thought that these results might be explained in two aspects of small sample in single trial and better tolerance in Chinese population. At last, transdermal fentanyl takes 12-24 hours for serum levels to stabilize after starting the patch and dose increment was trouble in clinic practice, so it is less flexible and needs to be used with caution in patients with unstable pain.

## Conclusions

In summary, the present study supported that transdermal fentanyl and sustained-release oral morphine were effective for maintenance therapy of moderate-severe cancer pain in Chinese population, and the former might have less adverse effects. Our results were similar with European and American data, which might suggest that both of opioids have no race choose. In addition, our data suggested transdermal fentanyl might improve QOL more easily. Well-designed randomised control trials should be further conducted in this area.

## List of abbreviations

CBMdisc: Chinese Biomedical Literature Analysis and Retrieval System; CNKI: China National Knowledge Infrastructure; VIP: Chongqi VIP Information; QOL: quality of life; RR: risk ratio; SE: standard error; WHO: World Health Organization; NCCN: National Comprehensive Cancer Network; IPD: individual patient data.

## Competing interests

The authors declare that they have no competing interests.

## Authors' contributions

DRX and QY contributed to the conception and design of the study; QY, DRX, and ZMJ contributed to collection and assembly of data; DRX, QY, ZMJ, WM, YDZ, ZFB, and DLC contributed to data analysis and interpretation; QY and DRX contributed to manuscript writing. All authors have read and approved the final manuscript.

## Supplementary Material

Additional file 1 **Characteristic of Eligible Cohort Studies**. Click here for file

Additional file 2 **Forest plots**. Click here for file
