# Supplementary material for: Efficacy and adverse effects of transdermal fentanyl and sustained-release oral morphine in treating moderate-severe cancer pain in Chinese population: a systematic review and meta-analysis
Source: J Exp Clin Cancer Res. 2010 Jun 9;29(1):67. doi: 10.1186/1756-9966-29-67 (PMC2904719; doi:10.1186/1756-9966-29-67)
Supplement: Additional file 1 — Characteristic of Eligible Cohort Studies. [file 1756-9966-29-67-S1.DOC]

**Additional file 1-Characteristic of Eligible Cohort Studies**

| Studies | Groups | No.of Patients | Quality score | Remission rate | Adverse effects | | |
| --- | --- | --- | --- | --- | --- | --- | --- |
|  |  |  |  |  | Constipation | Nausea/Vomiting | Vertigo/Somnolence |
| Cao YK ^8^ | Fentanyl  Morphine | 40  80 | 5 | 95%  97.5% | 25%  42.5% | 15%  27.5% | 0  12.5% |
| Dong HY ^9^ | Fentanyl  Morphine | 35  35 | 5 | 88.6%  91.4% | 8.6%  28.6% | 8.6%  14.3% | 22.9%  40% |
| Jiang B ^10^ | Fentanyl  Morphine | 28  28 | 5 | NR ^a^  NR ^a^ | 21.4%  71.4% | 17.9%  25% | 14.3%  21.4% |
| Jin BW ^11^ | Fentanyl  Morphine | 36  33 | 5 | 75%  75.8% | 16.7%  48.5% | 16.7%  48.5% | 13.9%  18.2% |
| Li R ^12^ | Fentanyl  Morphine | 40  40 | 5 | 80%  82.5% | 10%  72.5% | 32.5%  57.5% | 32.5%  32.5% |
| Meng LH ^13^ | Fentanyl  Morphine | 31  30 | 5 | 71%  73.3% | 16.1%  53.3% | 6.5%  33.3% | NR ^a^  NR ^a^ |
| Shen  J ^14^ | Fentanyl  Morphine | 40  40 | 5 | 90%  90% | 5%  25% | 25%  100% | 35%  90% |
| Wang X ^15^ | Fentanyl  Morphine | 34  33 | 5 | 94.1%  93.9% | 14.7%  63.6% | 26.5%  69.7% | 29.4%  18.2% |
| Wu  JH ^16^ | Fentanyl  Morphine | 52  52 | 5 | NR ^a^  NR ^a^ | 23.1%  34.6% | 21.2%  36.5% | 11.5%  19.2% |
| Zhang SJ ^17^ | Fentanyl  Morphine | 43  38 | 5 | 93%  86.8% | 16.3%  31.6% | 25.6%  57.9% | 2.3%  5.3% |
| Lei  W ^18^ | Fentanyl  Morphine | 32  35 | 5 | 68.8%  71.4% | 21.9%  48.6% | 15.6%  28.6% | NR ^a^  NR ^a^ |
| Guo  JP ^19^ | Fentanyl  Morphine | 26  37 | 5 | 92.3%  89.2% | 15.4%  37.8% | 11.5%  35.1% | 0  5.4% |
| Guo YW ^20^ | Fentanyl  Morphine | 38  38 | 5 | 81.6%  86.8% | 15.8%  65.8% | 7.9%  23.7% | 13.2%  10.5% |
| Li  JB ^21^ | Fentanyl  Morphine | 110  95 | 5 | 91.8%  89.5% | 7.3%  45.3% | 30%  30.5% | 4.5%  16.8% |
| Qu  YH ^22^ | Fentanyl  Morphine | 82  83 | 5 | 69.5%  72.3% | 17.1%  36.1% | 15.9%  25.3% | NR ^a^  NR ^a^ |
| Wu  B ^23^ | Fentanyl  Morphine | 30  29 | 5 | 80%  79.3% | 20%  48.3% | 20%  55.2% | 3.3%  3.4% |
| Yang  L ^24^ | Fentanyl  Morphine | 30  30 | 5 | 90%  93.3% | 20%  60% | 23.3%  30% | 16.7%  26.7% |
| Zhang JW^25^ | Fentanyl  Morphine | 23  27 | 5 | 95.7%  92.6% | 8.7%  77.8% | 60.9%  85.2% | 47.8%  51.9% |
| An  HZ ^26^ | Fentanyl  Morphine | 24  32 | 5 | 79.2%  81.3% | 16.7%  53.1% | 25%  46.9% | 45.8%  75% |
| Bai  Y ^27^ | Fentanyl  Morphine | 12  27 | 4 | 83.3%  88.9% | 25%  63% | 16.7%  26% | 41.7%  70.4% |
| Jin  XJ ^28^ | Fentanyl  Morphine | 30  48 | 5 | 90%  95.8% | 23.3%  62.5% | 40%  62.5% | 13.3%  10.4% |
| Lan HT ^29^ | Fentanyl  Morphine | 31  37 | 4 | 83.9%  86.5% | 22.6%  62.2% | 16.1%  24.3% | 51.6%  70.3% |
| Li  RM ^30^ | Fentanyl  Morphine | 30  28 | 4 | 83.3%  89.3% | NR ^a^  NR ^a^ | NR ^a^  NR ^a^ | NR ^a^  NR ^a^ |
| Li  ZB ^31^ | Fentanyl  Morphine | 42  50 | 5 | 90.5%  94% | 31%  64% | 35.7%  80% | 11.9%  10% |
| Lian ZP ^32^ | Fentanyl  Morphine | 38  30 | 5 | 84.2%  86.7% | 10.5%  63.3% | 13.2%  20% | 10.5%  56.7% |
| Liu  XF ^33^ | Fentanyl  Morphine | 27  31 | 4 | 81.5%  87.1% | 25.9%  29% | 18.5%  19.4% | 48.1%  90.3% |
| Pang DM ^34^ | Fentanyl  Morphine | 68  56 | 5 | 100%  100% | 14.7%  37.5% | 26.5%  69.6% | 4.4%  12.5% |
| Tang CR ^35^ | Fentanyl  Morphine | 67  75 | 5 | 89.6%  93.3% | 9%  24% | 47.8%  40% | 10.4%  8% |
| Wang GS ^36^ | Fentanyl  Morphine | 42  47 | 5 | 90.5%  91.5% | 7.1%  46.8% | 31%  34% | 14.3%  31.9% |
| Wang QC ^37^ | Fentanyl  Morphine | 54  32 | 5 | 92.6%  90.6% | 75.9%  90.6% | 22.2%  40.6% | 3.7%  28.1% |
| Yi  JQ ^38^ | Fentanyl  Morphine | 40  44 | 5 | 87.5%  95.5% | 15%  31.8% | 30%  40.9% | 10%  22.7% |
| Zhou ZJ ^39^ | Fentanyl  Morphine | 41  35 | 5 | 90.2%  88.6% | 7.3%  42.9% | 26.9%  34.3% | 24.4%  31.4% |

Toxicity profiles were reported according to the WHO’s criteria. Data in the table stood for incidence of all degrees of toxicity.

^a^ data couldn’t be extracted from original article.
