# Supplementary material for: Efficacy and adverse effects of transdermal fentanyl and sustained-release oral morphine in treating moderate-severe cancer pain in Chinese population: a systematic review and meta-analysis
Source: J Exp Clin Cancer Res. 2010 Jun 9;29(1):67. doi: 10.1186/1756-9966-29-67 (PMC2904719; doi:10.1186/1756-9966-29-67)
Supplement: Additional file 2 — Forest plots. [file 1756-9966-29-67-S2.DOC]

**Additional file 2**

a. Forest plot for pooled RR of pain remission.

b. Forest plot for pooled RR of constipation.

c. Forest plot for pooled RR of nausea/vomiting.

d. Forest plot for pooled RR of Vertigo/somnolence.
